# Assessing the Availability of Teleconsultation and the Extent of Its Use in Malaysian Public Primary Care Clinics: Cross-sectional Study

**DOI:** 10.2196/34485

**Published:** 2022-05-09

**Authors:** Sock Wen Ng, Wen Yea Hwong, Masliyana Husin, Norazida Ab Rahman, Nazrila Hairizan Nasir, Kawselyah Juval, Sheamini Sivasampu

**Affiliations:** 1 Institute for Clinical Research National Institutes of Health Ministry of Health Malaysia Shah Alam Malaysia; 2 Julius Center for Health Sciences and Primary Care University Medical Center Utrecht Utrecht University Utrecht Netherlands; 3 Family Health Development Division Ministry of Health Malaysia Putrajaya Malaysia

**Keywords:** teleconsultation, telemedicine, video consultation, telephone consultation, virtual clinic, primary care, cross-sectional, virtual care, Asia

## Abstract

**Background:**

The integration of teleconsultation into health care systems as a complement to existing approaches to care is growing rapidly. There is, however, limited information on the extent of its implementation across low- and middle-income countries.

**Objective:**

The aim of this study was to determine the availability and the extent of teleconsultation in Malaysian primary care clinics.

**Methods:**

A cross-sectional study of public primary care clinics in Malaysia was conducted between November 2020 and December 2020. All clinics in Malaysia that see more than 300 daily patients were recruited. A web-based, self-administered questionnaire including questions on availability of the service, whether it uses video or telephone, and the types of services it provides was distributed to the medical officer in charge of each clinic.

**Results:**

In total, 97.6% (249/255) of the clinics responded. Out of these clinics, 45.8% (114/249) provided teleconsultation. A majority of the clinics providing consultation (69/114, 60.5%) provided only telephone consultation, while 24.6% (28/114) of the clinics offered video and telephone consultation, and 14.9% (17/114) offered only video consultation. Eighty percent (92/114) of the clinics were located in urban areas. A breakdown by state showed that 17.5% (20/114) and 16.7% (19/114) of the clinics were from two larger states; other states comprised less than 10% each (range 7-9/114). For the clinics providing video consultation, funding for the service came mostly (42/45, 93%) from the Ministry of Health. Conversely, nearly 1 out of 4 (23/97) clinics that provided telephone consultation funded the service either from donations or through self-funding. Most of the clinics provided teleconsultation for diabetes and hypertension. Less than 50% of the clinics with teleconsultation used it for follow up with allied health care providers or pharmacists (video consultation, 20/45; telephone consultation, 36/97).

**Conclusions:**

Our findings show that telephone consultation is more widely used than video consultation, despite a quarter of its funding being self-subsidized or obtained through donations. Also, teleconsultation was less utilized by allied health care providers and pharmacists. Plans for the expansion of teleconsultation in Malaysian primary health care should take into consideration these findings to ensure a better and more cost-effective implementation of the service.

## Introduction

Telemedicine focuses on the use of information and communication technologies, such as computers, cell phones, and the internet, to provide clinical services remotely, to achieve long distance clinical health care [1], and subsequently to improve the overall efficiency of the health care system [2]. In an umbrella review of countries in the Organization for Economic Co-operation and Development, 83% of reviews found that telemedicine was as effective as face-to-face care, while 39% found that the use of telemedicine was cost-effective [3]. The use of telemedicine has also been reported to lead to high patient satisfaction. Nevertheless, in many low- and middle-income countries, comprehensive evaluations of the clinical and cost-effectiveness of telemedicine, as compared to conventional health care, have yet to be conducted. This is likely as a result of the poorer implementation of such services.

Despite the evidence, telemedicine has only been adopted on a large, global scale recently. A report from the World Health Organization showed that the proportion of countries with established telemedicine services ranged from 13% to 33% [1]. It also showed that telemedicine was provided more in high-income countries than in countries of other income statuses [1]. High-income countries that have implemented telemedicine programs include the United States [4], the United Kingdom [5], and various countries in Europe [6]. While high-income countries often face problems surrounding patient privacy and confidentiality, competing health system priorities, reimbursement, and infrastructure [1,3,7], the implementation of these services in low- and middle-income countries has been limited mainly by financial and technology infrastructure constraints [8]. For instance, the uptake of telemedicine in Pakistan, India, and Sri Lanka has been low, with the estimation that about 99.9% of the need for telemedicine remains unmet across these countries [9]. In particular, the integration of telemedicine services into primary health care settings as a complement to existing modes of care has also been slow [10].

Malaysia is one of the fastest-growing countries within Southeast Asia and is an upper-middle income country, with a per-capita income of RM 46,524 (US $11,512) [11]. In Malaysia, the first telemedicine blueprint was launched in 1997 [12] and was incorporated by the government into 1 of 7 flagship applications under the Multimedia Super Corridor project [13]. The government eventually established 4 main pilot projects, of which 1 involved teleconsultation between doctors of different disciplines and different health care facilities to overcome the lack of specialist care in rural areas [13]. In September 2019, the Ministry of Health (MOH) of Malaysia piloted teleconsultation based on video consultation technologies at 5 public primary care clinics in an effort to improve accessibility to health services and to reduce congestion at these clinics [14]. Bookdoc (Health4U Solutions Sdn Bhd), the current main platform contracted by MOH Malaysia for teleconsultation services, uses the fully Health Insurance Portability and Accountability Act (HIPAA)–compliant tool Twilio (Twilio Inc). Due to the COVID-19 pandemic, the teleconsultation service was expanded to an additional 35 public primary care clinics by the end of 2020. At the same time, many other clinics that were not part of the government initiative also proactively initiated teleconsultation in response to the pandemic. Despite such a recent rapid expansion of teleconsultation services, there is, at present, limited information on the extent and the availability of teleconsultation in primary health care settings in Malaysia.

Therefore, the objective of our study was to determine the availability and extent of teleconsultation in public primary care clinics in Malaysia. The focus of our study was only on synchronous teleconsultation in the form of video or telephone consultation between health care providers and patients, as part of either government projects or self-initiated projects. The information collected will be crucial to map out teleconsultation availability in Malaysia and help the MOH plan further expansion of the teleconsultation project in Malaysian primary care clinics.

## Methods

### Setting and Study Population

Primary health care in Malaysia is provided by both public and private health care providers. The MOH is the largest health care provider in Malaysia. The public sector is tax-funded, while the private sector is funded through fees for services, private health insurance, and employers, as part of employee health benefits [15,16]. Private primary care clinics are mainly located in urban and suburban areas, while public primary care clinics cover a wider area, including rural and remote areas [15]. Public primary care clinics under the MOH are classified into Types I, II, III, IV, V, and VI according to the total patient attendance per day [17]. Type I clinics have the greatest number of patients at more than 800 per day, while type VI has the lowest number of patients at less than 100 per day. In 2019, there were 1016 public primary care clinics led by medical officers or family medicine specialists [18]. In this study, we included all MOH public clinics categorized as Types I, II, and III.

### Questionnaire and Data Management

Consistent with our objectives, we developed a questionnaire to capture 3 aspects of teleconsultation: availability and whether it was based on video or telephone. In total, 15 questions were adapted from literature reviews and the local teleconsultation guidelines [19]. The questionnaire was pretested on 3 medical officers serving in public primary care clinics; each respondent was debriefed immediately after completion of the survey. A quick interview with the respondents was conducted to assess the comprehensibility of the questionnaire and whether the number of questions placed a burden on the taker. Modifications to the questionnaire were subsequently made in accordance with the pretest findings.

Study data were collected and managed using Research Electronic Data Capture (REDCap) hosted at the Clinical Research Centre, Penang General Hospital, Malaysia. REDCap is a secure, web-based software platform designed to support data capture for research studies. It provides (1) an intuitive interface for validated data capture; (2) audit trails for tracking data manipulation and export procedures; (3) automated export procedures for seamless data downloads to common statistical packages; and (4) procedures for data integration and interoperability with external sources [20,21].

### Data Collection

Data collection was conducted from November 6, 2020, to December 10, 2020. The study information sheet and survey link were sent to all state health departments along with an endorsement letter by the Family Health Development Division of the MOH before being distributed to the respective district health offices and selected clinics. Consent for participation was indicated by completion of the survey.

To improve the response rate for this survey, as the participation for this self-administered survey was voluntary and without compensation, we sent reminders to the participants who did not complete the survey 3 weeks after receiving the study invitation.

### Statistical Analysis

Descriptive analyses were conducted with continuous variables presented as the mean, median, SD, or IQR, while categorical variables were presented as frequencies and percentages. Analysis was performed using RStudio (version 1.3.1093; R Foundation).

### Ethics Approval

This study was approved by the Medical Research and Ethics Committee, Ministry of Health Malaysia (NMRR-20-1819-56089) with a waiver for informed consent, as the data collected were aggregated from each clinic without collection of any personal identifiers.

## Results

### Principal Outcomes

In total, 249 Type I to Type III clinics completed the questionnaire (for a response rate of 249/255, 97.6%). Of 13 states and 3 federal territories, 11 had a 100% response rate (Perlis, 4/4; Kedah, 18/18; Perak, 20/20; Melaka, 13/13; Negeri Sembilan, 16/16; Pahang, 11/11; Kelantan, 16/16; Terengganu, 12/12; Sabah, 12/12; Sarawak, 14/14; and Labuan, 1/1), while the remaining 4 states had response rates of 88% to 96.6%. Teleconsultation was provided in 114 (45.8%) of the public primary care clinics. The majority of these clinics (60.5%) provided only telephone consultation, followed by 24.6% (28/114) that offered video and telephone consultation, while 14.9% (17/114) had only video consultation. Of the remaining clinics that did not provide teleconsultation, 39.3% (53/135) planned to initiate the service.

Table 1 shows the characteristics of the clinics providing teleconsultation in Malaysia. In terms of distribution of the clinics across states in Malaysia, Selangor and Johor had the highest proportion, contributing to 17.5% (20/114) and 16.7% (19/114) of the total clinics providing the service, respectively. Other states accounted for less than 10% each; none were from Labuan. The majority of the clinics that offered teleconsultation were in urban areas (92/114, 80.7%). In addition, 69% (31/45) of the clinics that offered video consultation were Type I clinics. Interestingly, more than half of the clinics (38/69) that offered only telephone consultation were Type III clinics.

**Table 1:** Characteristics of the clinics.

Characteristics			Total (N=114)		Video consultation only (n=17)		Telephone consultation only (n=69)		Both video and telephone consultation (n=28)
**State, n (%)**									
	Johor	19 (16.7)		1 (6)		9 (13)		9 (32)	
	Kedah	7 (6.1)		0 (0)		6 (9)		1 (4)	
	Kelantan	10 (8.8)		1 (6)		8 (12)		1 (4)	
	Labuan	0 (0)		0 (0)		0 (0)		0 (0)	
	Melaka	9 (7.9)		1 (6)		7 (10)		1 (4)	
	Negeri Sembilan	7 (6.1)		2 (12)		4 (6)		1 (4)	
	Pahang	2 (1.8)		1 (6)		0 (0)		1 (4)	
	Penang	7 (6.1)		1 (6)		4 (6)		2 (7)	
	Perak	4 (3.5)		2 (12)		2 (3)		0 (0)	
	Perlis	4 (3.5)		0 (0.0)		4 (6)		0 (0)	
	Sabah	5 (4.4)		2 (12)		1 (1)		2 (7)	
	Sarawak	7 (6.1)		1 (6)		3 (4)		3 (11)	
	Selangor	20 (17.5)		3 (18)		14 (20)		3 (11)	
	Terengganu	4 (3.5)		1 (6)		3 (4)		0 (0)	
	WPKL and Putrajaya^a^	9 (7.9)		1 (6)		4 (6)		4 (14)	
**Location, n (%)**									
	Rural	22 (19.3)		3 (18)		14 (20)		5 (18)	
	Urban	92 (80.7)		14 (82)		55 (80)		23 (82)	
**Type of clinic^b^, n (%)**									
	Type I	44 (38.6)		12 (71)		13 (19)		19 (68)	
	Type II	29 (25.4)		3 (18)		18 (26)		8 (29)	
	Type III	41 (36.0)		2 (12)		38 (55)		1 (4)	

^a^WPKL and Putrajaya: Wilayah Persekutuan Kuala Lumpur and Putrajaya.

^b^The type of clinic was classified according to attendance numbers of patients per day: Type I, at least 800 patients per day; Type II, 500-800 patients per day; Type III, 300-500 patients per day.

### Characteristics of Teleconsultation Service

As shown in Table 2, telephone consultation had been offered for a longer time, with a median duration of 237 days, compared to video consultation, with a median of 107 days. Funding for video consultation in the clinics providing the service was mostly from the MOH (42/45, 93%). In comparison, nearly 1 out of 4 (23/97) clinics that provided telephone consultation service funded the service either by donation or through self-funding. Among the most frequent platforms used for video consultation were BookDoc (35/45, 75%), an online health care platform that supports video calls, followed by WhatsApp (9/45, 20%). For telephone consultation, about half the clinics (46/97, 47%) reported that they were using the health care provider’s own mobile phone.

**Table 2:** Characteristics of the services.

Characteristics of the service		Video consultation (n=45)	Telephone consultation (n=97)
Duration the service had been offered in days, median (IQR)		107.0 (91.0-121.0)	237.0 (182.5-288.0)
**Source of funding, n (%)**			
	MOH^a^ only	38 (84)	43 (44)
	MOH and self-funded	4 (9)	21 (22)
	MOH and donations	0 (0)	4 (4)
	MOH, donations, and self-funded	0 (0)	6 (6)
	Self-funded only	3 (7)	19 (20)
	Donations only	0 (0)	3 (3)
	Self-funded and donations	0 (0)	1 (1)
**Platform used^b^, n (%)**			
	Bookdoc	35 (78)	N/A^c^
	Whatsapp videocall	9 (20)	N/A
	Skype for Business	6 (13)	N/A
	Other^d^	5 (11)	N/A
**Type of device used, n (%)**			
	Clinic's landline/mobile phone	N/A	51 (53)
	Staff mobile phone	N/A	10 (10)
	Clinic's landline/mobile phone and staff mobile phone	N/A	36 (37)

^a^MOH: Ministry of Health Malaysia.

^b^Some clinics used more than one platform for video consultation, so the percentage sums to more than 100%.

^c^N/A: not applicable.

^d^Other platforms included Facebook video calls, Google Meet, GoToWebinar, Zoom, and Skype.

### Types of Health Care Services Provided Via Teleconsultation

**Figure 1:**
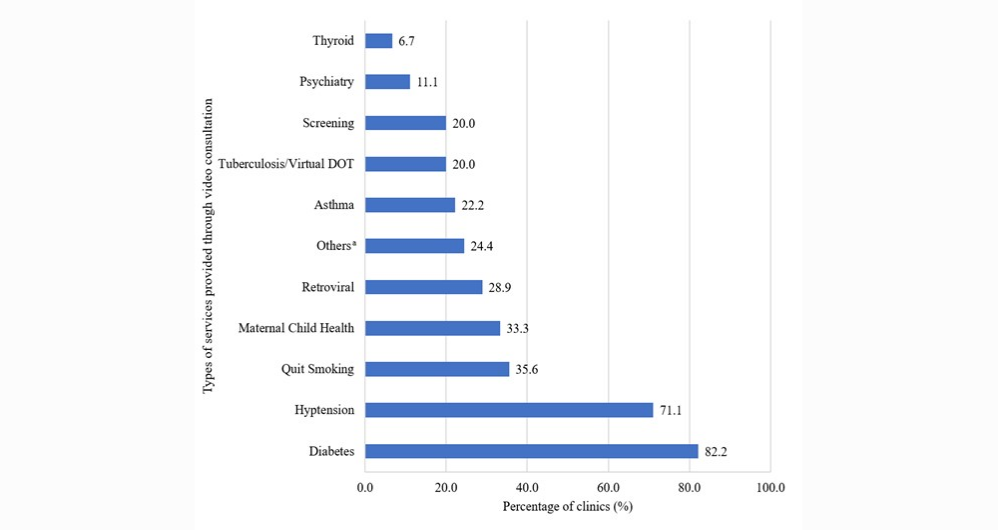
Type of services provided through video consultation. Some clinics provided more than one type of service using video consultation, so the percentages sum to more than 100%. a: Others included pre-pregnancy care clinic, obesity clinic, general outpatient consultation, and unspecified allied health service.

**Figure 2:**
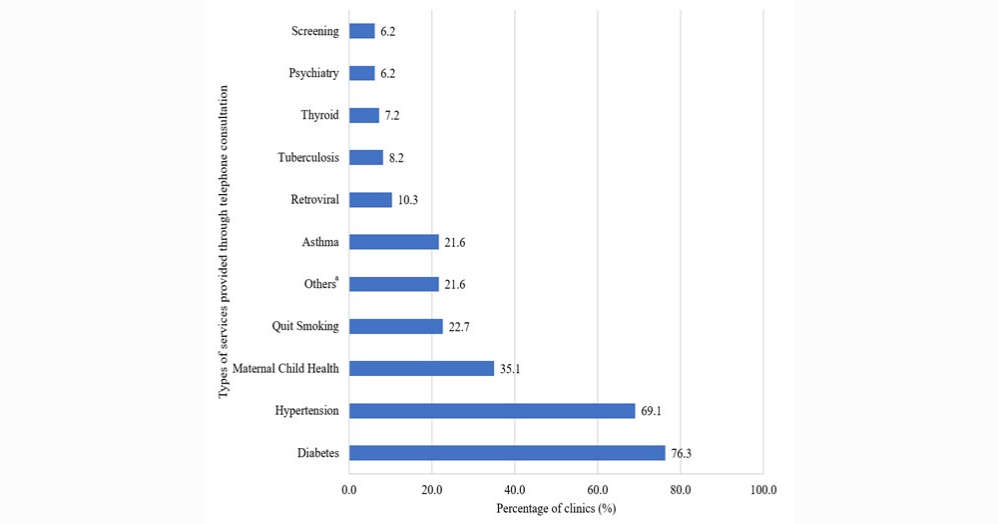
Types of services provided through telephone consultation. Some clinics provided more than one type of service using telephone consultation, so the percentages sum to more than 100%. a: Others included sexually transmitted infections, pre-pregnancy care, methadone, COVID-19 surveillance, one-stop addiction service, general outpatient consultation, unspecified allied health service, and unspecified pharmacist service.

Figure 1 and Figure 2 show the types of health care services provided by video and telephone consultation, respectively, at the clinics. The majority of clinics implemented video and telephone services for diabetes (37/45, 82% and 74/97, 76%, respectively) hypertension (32/45, 71% and 67/97, 69%, respectively), quitting smoking (16/45, 36% and 22/97, 23%, respectively), and maternal and child health (15/45, 33% and 34/97, 35%, respectively). Other services were also provided via teleconsultation, but in smaller proportions.

As demonstrated in Table 3, care plan consultation and health education were the most frequently provided services through teleconsultation, both via video and telephone. Less than half of the clinics utilized video or telephone consultation for follow up with allied health care professionals (20/45, 44% for video and 36/97, 37% for telephone). Furthermore, only 11% (5/45) of the clinics provided follow up with pharmacists via video consultation, while 5% (5/97) did so via telephone consultation. Virtual directly observed therapy for tuberculosis patients was provided via video consultation at 16% (7/45) of the clinics.

**Table 3:** Types of health care activities provided through teleconsultation.

Type of health care activity, n (%)	Video consultation (n=45), n (%)	Telephone consultation (n=97), n (%)
Care plan consultation	40 (89)	72 (74)
Health education	40 (89)	69 (71)
Disclosure of lab results	39 (87)	58 (60)
Follow up with allied health care	20 (44)	36 (37)
Follow up with pharmacist	5 (11)	5 (5)
Virtual directly observed therapy	7 (16)	N/A^a^

^a^N/A: not applicable.

## Discussion

### Principal Results

This is the first study attempting to map out the availability and extent of teleconsultation in Malaysian public primary care clinics. Our study found that 45.8% (114/249) of the clinics provided teleconsultation, with telephone being the most frequently used type (97/114). The majority of the clinics providing teleconsultation were in urban areas across Malaysia. Funding from the MOH was mostly used for the provision of video consultation in selected clinics, despite telephone consultation being more widely used. Less than half of the clinics with teleconsultation utilized the service for follow up with either allied health care professionals or pharmacists.

### Comparison With Prior Work

We found that less than half of public primary care clinics in Malaysia provided teleconsultation. In comparison, high-income countries have reported much higher proportions of the availability of this service. A cross-sectional study conducted in Norway showed that 80.8% of general practitioners in the country offered video consultation in 2020 [22]. Similarly, in Australia in 2020, teleconsultation was used by 96% of general practitioners [23]. Literature on the availability of teleconsultation in low- and middle-income countries is limited and has mainly been reported in the form of pilot projects or as interventions for research purposes [9,24]. A majority of this literature highlights financial and technological constraints as the main implementation barriers in low- and middle-income countries in initiating and providing teleconsultation [9,25].

Our study demonstrated that most of the clinics providing teleconsultation were in urban areas. The majority of clinics were located in Selangor and Johor, which are the two central, major state economies in Malaysia, with 91.4% urbanization in the former and 71.9% in the latter [26]. Absolute poverty is low, with an incidence estimated at 1.7% in Selangor and 5.9% in Johor; both states show a lower incidence than the national average, which was 8.4% in 2020 [27]. These findings are parallel to findings from a cross-sectional study in the United States that showed that more than half of hospitals that provided outpatient teleconsultation were from urban areas [28]. Additionally, Martin et al [29] reported similar findings in their study; they showed that only 3.3% of rural primary care providers and 8.3% of rural hospitals were implementing teleconsultation. The discrepancy in the availability of teleconsultation between rural and urban areas could be attributed to the digital divide, which has been defined as “the growing gap between the underprivileged members of society, especially the poor, rural, elderly, and handicapped portion of the population who do not have access to computers or the internet; and the wealthy, middle-class, and young living in urban and suburban areas who have access” [30]. In essence, there is a gap in availability of teleconsultation between urban and rural dwellers.

Generally, Malaysia has been portrayed as a highly digitized nation, with 88.7% of Malaysian households having access to the internet in 2020 [31]. Based on the International Telecommunications Union Report, which measures different aspects of internet penetration, an average of 81.2% of the Malaysian population are internet users, compared to the global average of 73.6% across 82 reporting countries in 2018 [32]. The proportion of internet users who are above the age of 50 years was reported to have increased sharply, from 4.2% in 2012 to 16.0% in 2018, accounting for a 2.1% increase in this age group in the general population [32]. Over the same time span, the average age of internet users in Malaysia also increased, from 29.7 years to 36.2 years, suggesting a narrowing digital divide in terms of age [32]. However, the same report demonstrated that higher median household income corresponded to a higher broadband subscription rate (with a correlation coefficient ranging from 0.59 to 0.72, *P*<.001) [32]. Urban households report a higher median household income of RM 6561 (US $1624) compared to a rural median household income of RM 3828 (US $947) [33]. This further supports our finding that digital divide is widening between urban and rural populations in Malaysia. Furthermore, this observation corresponds to the results of the Malaysian Internet Users Survey 2020, which showed that 75.6% of internet users were from urban areas [34]. The same survey also reported that one-third of Malaysian internet users resided in Selangor or Johor [34]. Even though the prevalence of noncommunicable diseases in rural and urban areas is almost the same [35], the utilization of public health care facilities is higher among rural dwellers [36]. Therefore, this urban-rural divide works against the initial aim of developing teleconsultation, which was to allow health care providers to overcome health service accessibility issues, especially for rural patients [25].

It is also worth noting that compared to video consultation, telephone consultation was more widely used by the clinics, even though a quarter of the telephone consultations were self-subsidized or funded by donations. This finding is in line with Brant et al [37], who found that the majority of practices in 5 areas of the United Kingdom conducted telephone consultation, while none provided video consultation. In addition, Heba and colleagues [38] reported that during COVID-19, 96.6% of general practitioners in southwestern Ontario used teleconsultation, but 99.5% of this was conducted via telephone consultation. A study looking at telehealth implementation in Australia showed that even with a change in reimbursement policy in the country, Australian health care providers, especially primary care providers, still preferred telephone consultation over video consultation [39]. While there is a lack of literature looking at synchronous teleconsultation between patients and health care providers over either video or telephone in low- and middle-income countries, we speculate that the availability of video consultation would be similar in Malaysia. This could be due to barriers in the adoption of video consultation, such as infrastructure requirements, digital proficiency, cost, and technical support availability [40], which can be extremely challenging to overcome, especially in low- and middle-income countries. By contrast, telephone consultation has a low start-up cost [41] and is easier to implement [42]. In our present study, the majority of the clinics that used video consultation were public primary care clinics, which have been identified for inclusion in the virtual clinic initiative funded by the MOH. Cost is likely one of the main reasons for the preference for telephone consultation over video among primary care providers from clinics that were not included in the initiative. As observed in our results, some clinics provided both telephone and video consultation services. However, we were not able to identify the proportion of exclusively video consultations to video consultations that were eventually converted to telephone, nor the reasons for why these unsuccessful video consultations were converted to telephone.

Our study also reports that the majority of clinics provided teleconsultation services for patients with chronic diseases, such as diabetes mellitus and hypertension. Similar findings were made by Kim et al [43], who reported that diabetes mellitus and hypertension accounted for 39.4% of teleconsultations conducted in their clinics. With the high prevalence of noncommunicable diseases in Malaysia, such as diabetes (with a prevalence of 18.3% in 2019) and hypertension (prevalence of 30.0% in 2019) [35], the bulk of the workload in public primary care clinics has always been the management of these patients. Active use of teleconsultation as an alternative method of consultation for these groups of patients would reduce crowding in clinics without compromising continuity of care for patients. Moreover, there are potential opportunities to expand the services to accommodate telemonitoring of these patients. Both services have been shown to be useful to improve disease control while at the same time reducing the use of resources [44].

Another interesting finding from our study is that very few clinics used teleconsultation for follow up with allied health care professionals or pharmacists. This is in keeping with results from a previous study in Brazil that showed only 5.8% of total teleconsultation was conducted by allied health care professionals or pharmacists [45]. Other than infrastructure and technology constraints, the limited use by allied health care professionals could have been due to their perceptions and attitudes, as well as a lack of information and training [46,47]. Similarly to patients, allied health care professionals, particularly physiotherapists, perceive teleconsultation negatively due to concerns about subpar health service due to the lack of hands-on examination and the lack of equipment at home, an attitude that could affect the uptake of teleconsultation [46]. The same concern about hands-on care has also been expressed by occupational therapists [48]. Allied health care professionals and pharmacists play an important role in providing complete, comprehensive care in managing primary care patients. They usually provide services like counseling on medication adherence and dietary intake, physiotherapy, and occupational therapy sessions during follow up. These forms of health care service have been shown to be as effective when delivered via teleconsultation as face-to-face [49,50]. Thus, the use of teleconsultation should be encouraged among these groups of health care providers.

### Implications for Policy and Research

The primary care setting is the best place for the adoption of teleconsultation, because it is where management of chronic conditions largely takes place [16]. The COVID-19 pandemic has demonstrated the relevance of teleconsultation, as the need for routine clinic visits by patients with chronic diseases tended to decrease during the pandemic. Hence, teleconsultation appears poised to stay a robust option for primary care in the near future.

Our findings showed that as of the end of 2020, approximately 50% of public primary care clinics in Malaysia provided teleconsultation. Our findings also indicate that the rate of adoption of teleconsultation differs between urban and rural areas. Nevertheless, several factors can be addressed to increase the effective and successful spread of such services, as well as their scaling up.

First, policymakers have to identify the obstacles that hinder the efficient delivery of teleconsultation services in primary care. It is imperative to understand potential barriers in order to offer solutions that can enhance the rate of adoption of teleconsultation. For example, technology that is unreliable or a lack of access to technology and broadband internet may pose barriers to video visits. This in turn may prevent end users from participating in teleconsultation. Thus, to increase teleconsultation utilization, the focus should be on providing end users with instant, always-on access that can be used anywhere and at any time. Alternatively, telephone consultation or asynchronous teleconsultation might be a better option for areas that are not equipped to participate in synchronous video consultation.

Second, proactive efforts should be made to reduce disparities in access to health services for vulnerable populations with limited digital literacy or access to technology, such as rural residents, if teleconsultation is to be implemented nationwide. Access to technology or digital inclusion has been increasingly considered as a leading social determinant of population health. As such, strategies to narrow the digital divide are especially important for increasing the use of digital applications in health care, not only to help the uptake of teleconsultation in the country, but also, crucially, to promote health equity in the population [51,52]. This is also an important consideration in primary care, especially because clear communication between health care providers and patients is essential for successful management of chronic diseases.

Third, it is important to change health care providers’ behavior, especially that of allied health care professionals and pharmacists, regarding their willingness to use this technology for patient care. The successful adoption of teleconsultation could potentially overcome the issue of personnel shortages among allied health care professionals and pharmacists. For instance, there were only a total of 64 dietitians in Malaysia in 2019 serving patients in 1016 public primary care clinics in the country [18]. The use of teleconsultation would allow group dietary counseling to be provided simultaneously to multiple patients and to remote areas.

Fourth, additional research on the teleconsultation experiences of patients and health care providers is imperative to enable us to obtain sufficient information on some of our findings, for example, to explain why telephone consultation was more widely used, or why certain clinics decided to utilize both telephone and video consultation. It is also crucial to account for the views of patients on the perceived usefulness and feasibility of teleconsultation services. Targeted strategies for successful implementations can only be planned after understanding the factors influencing the use of teleconsultation services among health care providers in Malaysia.

### Limitations

To the best of our knowledge, our study is one of the first in low- and middle-income countries to provide vital information on the availability and the extent of synchronous teleconsultation services in a primary health care setting. This study has several limitations. First, this is a descriptive cross-sectional study, and it is not possible to determine the causality of the findings. Second, our study only involved public primary care clinics. Nevertheless, we are confident that our findings, to a certain extent, reflect a near-actual representation of teleconsultation services in Malaysian primary health care. This is mainly because we had a relatively high response rate; the public primary care clinics in this study cover 64.6% of all primary care attendance in Malaysia [36]. Third, we only examined synchronous teleconsultation between health care providers and patients; thus, our findings are only relevant to this target population.

### Conclusion

In summary, we found that the availability of teleconsultation in public primary care clinics in Malaysia remains inadequate, with telephone consultation being more widely used than video consultation. Furthermore, there was a low utilization of these services among allied health care professionals and pharmacists. Plans for the expansion of teleconsultation in Malaysian primary health care should take into consideration these findings to ensure a better and more cost-effective uptake of teleconsultation in this country.

## Abbreviations

MOH: Ministry of Health
